# Supplementary material for: Are carbon emissions trading and green financial instruments synergistic? -Comprehensive quantitative research based on content analysis
Source: PLoS One. 2024 Mar 7;19(3):e0298601. doi: 10.1371/journal.pone.0298601 (PMC10919601; doi:10.1371/journal.pone.0298601)
Supplement: S1 Data — (ZIP) [file pone.0298601.s001.zip › Data/地级市Z_F工作报告.zip!/╔╜╬≈/╠1⁄2╘¡╩╨/└┤╫╘╣1⁄2╓┌║┼í╛┬φ┐╦╩2╛▌═°í┐_2011.docx]

各位代表：

　　现在，我代表市政府向大会作工作报告，并就《太原市国民经济和社会发展第十二个五年规划纲要（草案）》作说明，请予审议，请政协委员和列席人员提出意见。

　　一、“十一五”时期及2010年国民经济和社会发展回顾

　　“十一五”时期是我市发展史上极不平凡的五年。五年来，面对国际金融危机的严重冲击，面对资源型经济转型发展和结构调整的迫切要求，面对保增长、保民生和保稳定的繁重任务，在省委、省政府和市委的坚强领导下，我们深入贯彻落实科学发展观，扎实工作，奋力进取，全市经济社会发展和各项工作都取得了新成就。

　　2010年，完成地区生产总值1778亿元，比上年增长11%；规模以上工业增加值达到577.7亿元，比上年增长15.4%；全社会固定资产投资916.5亿元，比上年增长17.2%；社会消费品零售总额825.9亿元，比上年增长18%；财政总收入321.9亿元，比上年增长15.1%；一般预算收入138.5亿元，比上年增长17.8%。以上六项指标分别是“十五”末的1.98倍、2.05倍、2.09倍、2.15倍、1.97倍和2.43倍，比“十五”末基本上翻了一番。2010年城市居民人均可支配收入达到17258元，比上年增长10.6%；农民人均纯收入7611元，比上年增长11.5%。这两项指标分别是“十五”末的1.65倍、1.73倍。

　　（一）经济结构调整全面推进，增长质量和效益得到新提高。

　　工业转型取得新进展。煤矿兼并重组如期完成，煤矿数量由130座减到53座；非煤矿山关闭整合工作成效明显，非煤矿山数量由221座压减到97座；冶金、装备制造等重点产业改造升级，太钢5万吨不锈钢无缝钢管等一批重大项目建成投产；新能源、电子信息等新兴产业迅速发展，太阳能薄膜电池、物联网应用等项目稳步推进。高新区、经济区、民营区和不锈钢园区提档扩容，保持了较快发展势头，初步形成和集中了一批在国内外颇具竞争力的产业集群。工业经济综合效益指标达到185.3，比“十五”末提高44.9。

　　农业产业稳步发展。按照统筹城乡发展的要求、发展现代都市农业的思路，加强无公害蔬菜、标准化养殖、优质粮三大产业基地建设。全市无公害农产品基地达到43.8万亩，粮食总产量稳定在28万吨左右。大力发展食醋、乳制品、干鲜果、蔬菜和肉制品五大加工业，各类农业产业化企业发展到185个，带动18万农户增收；12个企业或产品名列全国同行业第一。指导发展农民专业合作社1851个。农村经济总收入达到590亿元，较“十五”末增长40.5%。

　　第三产业快速增长。服务业成为拉动经济增长的重要力量，我市被确定为国家再生资源回收体系建设、标准化菜市场建设、家政服务体系建设、加工贸易梯度转移建设和国家物流园区试点城市，服务外包被列入全国基地城市。加快发展会展业，“能博会”规模不断扩大，成为在国内外有较大影响力的品牌。稳步推进新农村现代流通网络建设和“万村千乡市场工程”，新建、改建农家店、农资店2416个，乡镇覆盖率达到100%。文化产业有序推进，组织实施了 “五个一工程”。旅游业快速发展，国家4A级旅游景区达到7处，旅游总收入较“十五”末增长1.86倍。地区外贸进出口总额达到79.1亿美元，是“十五”末的2.35倍。

　　（二）节能环保与生态建设有效推进，环境友好型社会建设取得新进展。

　　节能降耗效果明显。淘汰460户企业的落后产能，对148户重点用能单位进行强制性能源审计，万元地区生产总值综合能耗下降27%。开工重点节能项目150个，可再生能源建筑应用面积达到550万平方米，我市成为国家第一批可再生能源建筑应用示范城市。建成循环经济示范项目10个，经济区获批“国家生态工业试验区” ，西山煤电荣获“联合国清洁煤示范企业”称号。

　　环境保护力度加大。严守“绿色高压线”，关停取缔落后生产设施930个，否决不符合产业政策的项目383个。污染减排任务超额完成，生活垃圾和生活污水处理能力得到提升，化学需氧量、二氧化硫排放量分别下降15.2%和32.9%。市区大气环境综合污染指数持续下降。城市集中式饮用水源地水质达标率保持100%。

　　生态建设成效显著。全市累计造林115.5万亩，城郊森林公园和环城林带建设快速推进。建成汾河景区二期等一批园林绿化工程，建成区绿化覆盖率达到36.8%，绿地率达到31.98%，人均公园绿地面积9.59平方米，我市获“国家园林城市”荣誉称号。治理水土流失面积102.3万亩，建设节水工程28处。大力推进西山地区综合整治，“万亩生态园”建设示范作用显著，西山国家矿山公园成功获批。

　　（三）城市承载能力有效提升，城镇化建设迈出新步伐。

　　城市规划、建设和管理全面推进。科学组织编制城市概念及发展规划、总体规划、专项规划和片区规划等200余项。强化基础设施建设，累计完成投资287.6亿元，完成滨河东路北延、祥云桥等建设项目413项；房地产开发投资累计完成702.4亿元。创新管理模式，实现建管分离，城乡管理的科学化、专业化水平得到提升。深入推进“城乡清洁工程”，实施“十项清洁创建”和“五项专项整治”，基本实现清洁体系城乡全覆盖。“治超”工作取得明显成效。

　　城镇化建设步伐加快。着眼于推进城乡一体化，以主城区发展、产业辐射带动周边小城镇发展。全市7个小城镇被列为全国重点小城镇，开工和建成一批建设项目。基本建立了城乡户籍一体化登记制度，为小城镇建设注入新活力。新农村建设快速推进，农村“五个全覆盖”提前实现。高标准开展“四化四改”，大力扶持农村新能源建设，受益农户达3.2万户。建设农村公路4400公里。建制村班车通达率100%。

　　（四）民生得到较好保障和改善，社会事业取得新发展。

　　切实保障和改善民生。积极开展创业型城市创建工作，城镇登记失业率始终控制在4%以内。社会保障体系建设取得突破性进展，城市养老保险覆盖面持续扩大，连续五年调高退休人员养老金待遇；在全省率先实行农村养老保险制度全覆盖；在全国率先实现农村“五保户”全部集中供养，率先实施社会散居孤儿最低养育制度；累计保障城市低保对象91.9万户次、农村低保对象72.3万户次。城镇职工、城镇居民和新农合三项医疗保险制度无缝对接，实现了“全民医保”。6.5万农村贫困人口实现脱贫，减幅48.2%。解决58.6万农村人口安全饮水问题。大力实施保障性住房建设，开工9.87万套，竣工3.94万套。降低公交票价，群众得到更多实惠。

　　社会事业全面发展。科技引领支撑作用不断增强，煤层气燃料重型卡车等一批高新技术项目成为新的经济增长极。出台4部地方性科技法规。全社会研究与试验发展经费投入、专利申请量分别是“十五”的6.5倍和4.6倍，科技成果转化率达到50.4%，高新技术产业增加值年均增长16%，我市被确定为国家创新型城市试点和国家知识产权示范市。实施中小学校舍安全工程与“百校兴学”工程，投资67.2亿元，改造校舍845所，新增优质学校162所，减少薄弱学校289所，我市校舍改造经验被命名为“太原模式”，在全国推广，全国校舍安全现场会在太原召开。获得“全国未成年人思想道德建设工作先进城市”荣誉。加强基层医疗卫生服务能力建设，县乡村三级医疗机构基础设施达标率提高47个百分点。群众体育蓬勃开展，获得“全国实施体育锻炼标准先进城市”荣誉；竞技体育成绩显著；成功承办女子摔跤世界杯团体赛、小轮车世锦赛、首届太原国际马拉松赛等重要赛事。繁荣文艺创作，推出晋剧《傅山进京》等一批艺术精品。新建、改扩建53个乡镇文化站，建成晋商博物馆等重要文化设施。连续两届获得“全国创建文明城市工作先进城市”荣誉。获得“全国人民防空先进城市”荣誉。实现“全国双拥模范城”六连冠。第六次人口普查取得阶段性成果。广播电视、新闻出版、人口计生、防震减灾、民族宗教、文物、气象、外事、地方志、档案工作取得新进展。老龄、残疾人、慈善救助和红十字事业取得新成绩。

　　（五）改革开放不断深化，发展环境有了新的改善。

　　重点领域改革不断深化。政府机构改革基本完成，事业单位分类改革进展顺利，人事制度改革迈出新步伐。深化财政体制和增值税转型改革，市对县财政管理体制实现创新。国企改革三年攻坚阶段目标基本完成，企业发展势头较好。文化体制改革有序推进，组建文广、报业、创意、新晋商、电影五大集团。

　　发展环境持续改善。改革行政审批制度，审批事项由889项减少到383项；实行基本建设项目“一票制”收费，推行外商投资企业“一表制”审批，建立“三位一体”政务督查机制。加大政务公开力度，成为“全国政务公开示范城市”。出台《绿色转型促进条例》，制定颁布绿色标准33个，成为全国第一个以地方立法形式整体推动绿色转型的城市、全国率先系统制定实施绿色标准的城市。出台关于创优发展环境、培育“绿色百强”、实现跨越发展的38条政策意见。

　　开放程度不断提高。民营经济增加值增长2.3倍，占全市地区生产总值的比重达到47.4%；新孵化民营企业5600家，从业人员净增7.5万人，总数占全社会的66%。狠抓重点项目推介及招商引资，积极组团参加“中博会”等国内重大经贸洽谈活动。新批设立外商投资企业177户，项目总投资65.2亿美元，实际利用外资12.3亿美元（部口径）。

　　（六）民主法治建设不断加强，安全稳定形势实现明显好转。

　　加强民主法治建设。自觉接受人大的法律监督和工作监督，主动接受政协的民主监督，积极支持各民主党派、工商联、无党派代表人士参政议政。五年来，共办理人大代表建议和政协提案3469件；向市人大常委会提请审议地方性法规草案28件。扎实推进“法治太原”建设，“五五”普法成效明显。强化行政监察和审计监督，行风政风逐步好转，廉政建设和反腐败斗争取得积极成效。

　　社会保持安全稳定。加强矛盾纠纷排查化解和群体性事件处置工作，社会保持稳定。加强和改进信访工作，加大重信重访案件处置力度，信访形势总体平稳。深入推进“晋阳风暴”严打整治专项行动，群众安全感不断提升。切实加强安全生产工作，安全生产形势明显好转。

　　各位代表，我向大会郑重报告，我市“十一五”规划目标任务较好地完成，向全市人民承诺的实事全部兑现！

　　回顾“十一五”时期的政府工作，我们深切地认识和体会到，办好太原的事情，必须把思想统一到中央、省委、省政府和市委的决策部署上来，坚定不移狠抓落实；必须深刻把握国内外发展形势，以转型发展为主线，以赶超发展为战略，以跨越发展为目标，抢抓机遇，加快发展；必须把保障和改善民生作为一切工作的出发点和落脚点，高度重视发展社会事业和改善民生，让人民群众共享改革发展成果；必须增强“对标”意识，树立“要干就干最好、要做就做第一”的思想，把太原放在全国省会城市大坐标系中找出差距，制定措施，奋力赶超；必须坚持紧紧依靠广大干部群众，凝聚各方智慧和力量，调动一切积极因素，同心同德，共谋发展。

　　各位代表，五年的成绩来之不易。这是省委、省政府和市委统揽全局、坚强领导的结果；是市人大、市政协依法监督、民主监督、大力支持的结果；是社会各界关心支持、共同努力的结果；是全市人民辛勤劳动、团结奋斗的结果。在此，我代表市政府，向全市人民、各民主党派、各人民团体和社会各界人士，向驻并部队、武警官兵和国家、省驻并单位，向所有关心支持太原发展的海内外朋友们，表示崇高的敬意和衷心的感谢！

　　我们也清醒地认识到，我市经济社会发展还存在着不少困难和问题：发展不足、投资不足、后劲不足的问题依然突出；结构调整、产业升级、增强竞争力任务艰巨；城市承载能力弱，节能减排压力大，生态环境仍然很脆弱；体制机制创新不够，发展环境还不宽松；社会事业和民生建设相对薄弱，社会管理和服务水平亟待提高，城乡居民收入不高、增长不快；政府职能转变相对滞后，部分干部的思想观念和工作作风与转型跨越发展的要求还不相适应。

　　我们将切实增强危机意识、忧患意识和责任意识，充分利用有利条件，不断采取有效措施，加快解决这些问题，绝不辜负全市人民的期望和重托。

　　二、“十二五”时期经济社会发展的指导思想和目标任务

　　“十二五”时期，是全面建设小康社会的关键时期，也是加快转型跨越发展的重要时期。这一时期，我市经济社会发展面临着难得的机遇：从国内外宏观环境看，科技创新和产业转型孕育新突破，有利于我市抓住机遇，提升产业素质，推进新兴产业的发展；太原城市群建设上升到国家战略层面，将使我市得到更多的发展空间。从全省看，省委、省政府大力支持太原率先发展，大力支持太原在“国家资源型经济转型综合配套改革试验区”建设中先行先试； “一核一圈三群”城镇化建设全面实施，有利于我们整合区域资源，获得政策支持。从我市自身看，不锈钢、煤机和重型装备制造、镁合金等产业优势突出，创新资源要素集中，东中西部交通枢纽和物流中心地位重要，使我们有条件加快发展速度，提高发展质量；特别是市委审时度势，提出把太原建成一流的省会城市，全市上下思想统一、目标一致、精神振奋，率先发展的氛围更加浓厚。

　　根据市委《关于制定国民经济和社会发展第十二个五年规划的建议》，我们编制了《太原市国民经济和社会发展第十二个五年规划纲要（草案）》，提交大会审议。

　　“十二五”经济社会发展指导思想：全面贯彻落实党的十七届五中全会精神和省委九届十一次全会精神，以邓小平理论和“三个代表”重要思想为指导，深入贯彻落实科学发展观，以科学发展为主题，以加快转变经济发展方式为主线，坚持把经济结构战略性调整作为主攻方向，坚持把科技进步和创新作为重要支撑，坚持把保障和改善民生作为根本出发点和落脚点，坚持把建设资源节约型、环境友好型社会作为重要着力点，坚持把改革开放作为强大动力，大力推进工业新型化、农业现代化、市域城镇化、城乡生态化，在全省转型跨越发展中发挥表率作用，把太原建成一流的省会城市，努力成为具有国际影响力的区域性现代化大都市。

　　“十二五”时期经济社会发展的主要目标是：

　　综合实力显著增强。地区生产总值年均增长13%，达到3600亿元；财政总收入、一般预算收入年均增长15%，分别达到648亿元、278亿元；全社会固定资产投资年均增长28%，累计完成1万亿元。

　　结构调整取得重大进展。以新兴产业为主体的产业体系基本形成，拉动经济增长的产业结构趋向合理，传统产业新型化率达到80%；自主创新能力显著提升，高新技术产业增加值占地区生产总值的比重达到20%。

　　城乡居民收入大幅增加。努力实现居民收入增长和经济发展同步，劳动报酬增长和劳动生产率提高同步，城镇居民人均可支配收入达到31800元，农民人均纯收入超过14000元。

　　城镇化水平显著提高。城市规模稳步扩大，建设水准明显提升，服务功能逐步完善，管理信息化、精细化水平切实提高，新城建设取得突破性进展。城乡发展协调性明显增强，城镇化率达到84%。

　　生态环境明显改善。单位地区生产总值能耗和主要污染物排放总量大幅下降，环境空气质量达到国家二级标准。东西两山生态恢复、绿化和汾河流域整治取得重大进展，森林覆盖率力争达到25%，城乡生态系统走上良性发展轨道。

　　社会建设进一步加强。覆盖城乡的基本公共服务体系日臻完善，城镇登记失业率控制在4%以内。社会主义民主法制更加健全，人民权益得到切实保障。社会管理创新取得实效，社会更加和谐稳定，安全生产形势稳定好转。城乡抵御各种自然灾害的能力进一步增强。

　　改革开放步伐不断加快。重要领域和关键环节改革取得突破性进展，资源型经济转型综合配套改革先行先试取得显著成效。政府职能进一步转变，执行力明显提高，发展环境显著改善。对外开放广度和深度持续拓展，开放型经济达到新水平，外贸进出口总额达到160亿美元。

　　“十二五”时期经济社会发展的主要任务是：

　　（一）全力推进产业新型化，建设一流的新兴产业基地。坚持走“新兴化、高端化、园区化、集群化、循环化和信息化”发展之路，建设全国一流的新兴产业基地。加快推进工业新型化，着力打造“三大产业集群”，着眼于抢占新兴产业竞争制高点，大力发展重型机械、矿用成套设备、铁路装备、汽车及零部件制造等产业，在成套化、系列化、标准化上下功夫，建设世界一流的“装备制造产业集群”；大力发展不锈钢、镁合金、高性能磁材等新材料产业，在深加工、终端产品化上下功夫，打造世界一流的“以不锈钢和镁合金为主的新材料产业集群”；大力发展新能源、节能环保、生物医药、信息技术等产业，在规模化、循环化上下功夫，打造全国一流的“高新技术产业集群”。加快发展现代服务业，着力打造“环渤海和黄河中游地区现代物流中心”、“华夏文明看山西的旅游中心”、“国际性的专业会展中心”、“区域性金融中心”。大力推进物流业、旅游业、会展业、金融业的创新与发展，大力发展生产性服务业，提升生活性服务业的水平。加快推进农业现代化，着力发展都市农业，加强新农村建设，大力发展县域经济，加大资源节约型和环境友好型农业建设力度，积极培育农产品加工龙头企业，提升农业产业化水平。

　　（二）加快实施科教兴市和人才强市战略，建设一流的自主创新基地。把科技进步作为转型跨越发展的重要支撑，加快自主创新步伐。充分发挥省城创新要素集中的优势，整合科技资源，搭建创新平台，优化创新环境，培育创新主体。加快完善科技创新体系，构建各类产业集群技术联盟和产学研战略联盟，实施战略性新兴产业培育等科技创新支撑转型跨越发展八大工程，提升产业核心竞争力，推动全市经济发展由资源驱动型、资本驱动型向创新驱动型转变，努力建成国家创新型城市。强化科技与金融结合，加快建立多元化投融资体系。继续实施“百校兴学”工程，全面提升教育质量和水平。大力实施人才优先发展战略，实施创新人才培育工程，吸引和培养更多的领军人才，为建设一流的自主创新基地提供人才智力保障。

　　（三）加快城市规模化发展和生态化建设，建设一流的现代宜居城市。结合西山地区综合整治和晋阳湖开发，在市区南部、汾河两岸建设组团式布局的国际一流新城，以新城建设带动老城、疏解老城、服务老城。老城改造以完善功能为重点，实施公交优先发展战略，加快地铁、快速公交和智能交通系统建设；加强基础设施建设，增加绿地面积，扮靓老城风貌，大幅提升城市宜居度。以创建全国环保模范城为目标，加快建设“气化太原”、“净化太原”，严格控制工业污染，大力推进城中村改造和集中供热工程，彻底解决大气污染问题，再造蓝天白云之城。以创建国家森林城市、国家生态园林城市和全国卫生城市为目标，全面加强城乡生态建设，大规模推进绿化工程和“城乡清洁”工程，加大河湖水系综合整治力度，保护省城水源地汾河水库，彻底改善水环境，再造青山绿水之城。

　　（四）加强以改善民生为重点的社会建设，切实让转型跨越成果惠及广大群众。实施积极的就业政策，千方百计促进就业创业。坚持“广覆盖、保基本、多层次、可持续”的方针，实现城乡居民社会保障全覆盖。大规模建设经济适用住房、廉租住房、公共租赁住房和限价商品房。加强公共卫生体系建设，实施“百院兴医”工程，推行国家基本药物制度，巩固和完善新型农村合作医疗制度，实现基本公共卫生服务逐步均等化。坚持“安全第一，预防为主，综合治理”的方针，强化安全生产工作。推进人防建设与城市建设融合式发展，人均人防避难场所面积接近全国平均水平。深入开展平安省城建设，努力破解影响社会安全稳定的体制机制问题，形成较为完善的管理体系，维护省城社会和谐稳定。

　　（五）全面深化改革开放，着力增强率先发展的动力与活力。全面开展“对标”活动，坚定不移地推进经济、政治、文化、社会等领域的改革，加快构建有利于率先发展的体制机制，营造更加开放的发展环境，激发全社会的创造活力。大胆探索资源型经济转型综合配套改革试验区各项改革，先行先试。推进文化体制改革，正确处理城市建设与历史文化遗产保护的关系，把历史文化名城这张名片保护好、建设好，推动文化大发展大繁荣，建设特色文化名城。深化国有企业改革，加大资产重组力度，推进国有经济战略布局调整，打造一批综合竞争实力强的大中型企业集团。加快财税体制改革，优化财政支出结构，提高财政资金使用效能。强化投融资改革，规范政府投资行为，拓宽民间投资领域和范围。实施非禁即入政策，大力支持非公有制经济发展。加强区域经济合作，加大招商引资力度，以开放合作促进转型跨越发展。推进政府管理创新，建设效能政府、法治政府、服务政府和廉洁政府。

　　各位代表，建设一流省会城市是时代发展的要求，是人民赋予的重任。我们坚信，有全市上下万众一心、扎实苦干，一个经济更加繁荣、社会更加和谐、人民更加幸福、山川更加秀美的新太原一定会展现在世人面前！

　　三、2011年工作任务

　　今年政府工作的总体思路是：全面贯彻落实党的十七届五中全会、省委九届十一次全会和市委九届七次全会精神，紧紧围绕建设一流省会城市、建设具有国际影响力的区域性现代化大都市的目标，在国家资源型经济转型综合配套改革试验区建设上先行先试，以 “三个一流、一个基础、四大支撑”为重点，抓住发展机遇，全力推进工业新型化、农业现代化、市域城镇化、城乡生态化，突出改善民生，深化改革开放，在太原率先发展上迈出坚实步伐，为实现“十二五”宏伟目标开好局、起好步。

　　今年全市经济社会发展的主要预期目标是：地区生产总值增长13%，全社会固定资产投资增长25%，规模以上工业增加值增长17%，社会消费品零售总额增长16%，财政总收入和一般预算收入均增长15%，城镇居民人均可支配收入增长10%，农民人均纯收入增长10%以上，城镇登记失业率控制在4%以内，居民消费价格总水平涨幅控制在 4%左右。

　　约束性指标是：万元地区生产总值能耗下降3.5%左右，万元生产总值二氧化碳排放量下降3.5%，化学需氧量、氨氮、二氧化硫、氮氧化物、烟尘和工业粉尘排放量均控制在省政府下达的目标任务内，万元工业增加值用水量下降5%。新建开工保障性住房19186套、169.1万平方米。

　　围绕上述目标，在全面做好各项工作的同时，重点抓好以下八个方面的工作：

　　（一）加快推进经济结构战略性调整，在构建现代产业体系上下功夫。

　　建设一流的新兴产业基地，必须加快经济结构调整，转变经济发展方式，推动经济尽快走上科学发展、转型跨越的道路。

　　着力推进工业新型化。以“三大产业集群”为主体，大力培育壮大有太原特色的战略性新兴产业。加大对高端装备制造、节能环保等产业的扶持力度，重点推进太原铁路装备造修基地、中国电科太原光伏产业园、阳煤集团新型化工材料园、富士康苹果产业园、新天地静脉产业园等项目建设。大力改造提升传统产业，重点推进煤炭资源整合保留矿井实现规模化、集约化、机械化和信息化发展。推动西山煤电、太钢、古交市、清徐县等在国家和省循环经济试点建设上取得新突破。支持太钢碳钢冷连轧、5万吨不锈钢无缝钢管、2万吨高强度精密带钢、兴能发电三期、古交新型煤化工和循环经济示范区等重点项目建设。

　　加快发展第三产业。以建设“四大中心”为主体，改造提升传统服务业，全力发展现代服务业和生产性服务业。抓好武宿物流中心、山西农产品展示交易中心、再生资源回收利用体系等项目建设，提升服务业的档次和规模。加强物联网应用及推广，把太原打造成物联网应用示范城市。加大直接融资力度，抓好太原股权交易市场建设及“新三板”在太原的设立工作。加大文化投资，发展动漫游戏产业，建设清徐文化产业园。以“唐风晋韵·锦绣龙城”为主题，打造太原晋商文化艺术周、清徐醋文化节和太原国际面食文化节等特色文化品牌。举办好“中部六省中国优秀旅游城市采风”活动。加快旅游咨询集散中心建设，整合旅游要素市场，完善景区功能，推动景区升级，促进旅游业快速发展。继续实施太原旅游“一卡通”惠民工程。

　　加快园区建设步伐。大力推动产业、企业、项目和要素向园区集聚，提升产业集中度和园区经济带动力。加快高新区新区建设，重点推进电子信息等五大特色产业项目发展。加快经济区产业集群化发展，打造以太原为中心的世界煤机等五大产业基地，推进国家生态工业试验区建设。着力建设民营区工业新区和现代物流示范区，围绕扶持横店工业园项目，大力引进带动性强的大企业和大项目，重点发展新兴产业。抓好不锈钢园区国际物流中心、钢铁交易中心重点项目的建设。

　　大力发展现代农业。认真落实中央1号文件精神，加强农田水利基础设施建设，为农业生产提供有力保障。坚持依法自愿有偿的原则，健全完善农村土地流转机制，推进规模化生产和集约化经营。打造一批集生产性、生活型和生态型于一体的高效农业示范区，实现农业的生产标准化、基地规模化和园艺观光化，大幅提高农业产出效益。把“一村一品”、“一县一业”作为推进农业现代化的重要抓手，实施“百园兴农”工程，规划建设一批食醋、畜（乳）、蔬菜、干鲜果和粮食加工园区。抓好 “菜篮子”工程，实施菜地最低保有量制度，落实“设施蔬菜百万棚”计划；建立生产供应配送体系，开展“农超对接”，畅通农产品运输绿色通道。新发展农民专业合作社100个，打造一批星级示范社。农业产业化年销售收入达到80亿元，同比增长14%；农村经济总收入达到637亿元，同比增长8%。

　　（二）切实加强环境保护和生态建设，在建设环境友好型城市上下功夫。

　　建设一流的现代宜居城市，必须按照“绿色、低碳、洁净、健康”的理念，做好提高空气质量、修复生态环境两篇大文章，努力建设生态文明。

　　大力推进环境治理。围绕建设“蓝天白云之城”，加快推进“供热全覆盖”步伐，年内市区集中供热率达到80%以上，主城区范围内城中村集中供热率大幅提高。加快建设“气化太原”，坚持政府主导、市场配置的原则，整体推进煤制天然气、过境天然气、煤层气、焦炉煤气“四合一”，年内城市气化率明显提高。加快建设“净化太原”，严把新上项目的环保关，加快污染企业的搬迁，控制污染物排放总量，主城区内绝不能新增任何一个污染企业。加大企业改造升级力度，瞄准国内一流的能耗和排放水平，年内实现“达标”计划全覆盖。以电厂脱硝为重点，大力推进污染减排。深入推进清洁生产，对重金属污染行业和产能过剩行业进行强制性清洁生产审核。全方位遏制扬尘污染。强化机动车尾气监督管理。市区空气质量二级以上天数实现稳步增加。以娄烦县、迎泽区创建环保模范县（城区）、经济区创建环保生态园区为突破口，力争用两到三年时间将我市建成省级环保模范城，并全面启动国家环保模范城创建工作。

　　着力推进生态建设。围绕建设“青山绿水之城”，以创建国家森林城市、国家生态园林城市为目标，抓好十大造林绿化工程，今年完成造林47万亩。开展“绿染龙城、花满并州”三年植绿大行动。抓好全民绿化工作，以市区为重点，广泛开展单位、厂区、庭院绿化，大力推进大企业“造绿”。出台支持鼓励政策，大力发展民营林业。基本完成集体林权制度主体改革。抓好碳汇造林项目、东西山绿化、三县一市水土保持林、生态防护林、经济林和城郊生态公园建设。积极推进林地林木认养工作。实施龙城公园等14项园林绿化工程，新建20个小游园。建成区绿化覆盖率、绿地率均增加1个百分点，人均公共绿地增加0.5平方米。治理水土流失面积20万亩。精心打造城市水系，继续治理美化汾河太原段，对市区13条边山支流开展截污整治，对玉门河等6条河道进行综合整治。市区水域功能区达标率力争达到60%以上。饮用水源地水质达标率稳定保持100%。

　　强力推进节能工作。以开展国家循环经济标准化试点为重要抓手，完成一批循环经济示范项目建设。在工业领域推广循环流化床锅炉、余热余气瓦斯发电等节能产品和项目；在建筑领域推广以太阳能为主体的光电、光热一体化技术；在农村推广和实施沼气、秸秆气化和节煤省柴灶连炕技术。加强重点耗能企业节能管理，突出抓好100户耗能大户、40户煤焦和煤炭洗选企业。加大节能执法和节能审计力度，强制执行能耗限额标准。坚决执行固定资产投资项目节能评估和审查制度，严控高耗能行业。继续淘汰落后产能，压缩焦化产能30万吨。既有建筑节能改造150万平米，可再生能源开发利用100万平米。

　　推进西山地区转型发展和综合治理。以“城市扩容、土地增效、生态修复、产业升级”为目标，完成重点区域规划。高标准推进绿化造林，两年内实现绿化全覆盖，构建西山绿色长廊。年内关停淘汰区域内落后产能企业。积极支持中国北车集团建设铁路装备工业园、中国建材集团建设新型建材工业园、山西国信建设森林公园、太钢建设森林公园、西山煤电建设国家矿山公园等项目。启动晋阳湖水生态修复工程，加快推进采煤沉陷区治理和长风西大街启动区建设。

　　各位代表，冬季大气污染是影响全市环境质量的重症。今年我们将下大决心对城中村进行改造，下大决心大幅度提高市区集中供热率，下大决心抓好污染企业的关停限产，确保冬季大气质量明显改善，让全市人民呼吸到更加清新的空气，享受到更多的蓝天白云和青山绿水！

　　（三）统筹提升城乡基础设施建设和管理水平，在提高城市承载能力上下功夫。

　　建设一流的现代宜居城市，必须统筹城乡基础设施建设，提高管理和服务水平，增强城市综合承载能力。

　　高标准编制新城建设与老城改造规划。按照“以现代化为基干、以特色化为神韵、以人性化为根本，彰显现代都市风貌、体现国际影响力气魄”要求，上半年高标准编制完成太原南部新城发展战略规划、重点地段和片区规划，下半年启动新城建设。编制太原都市圈战略规划以及产业发展、生态布局、公共安全、综合交通等专项规划。完善市区范围内的控制性详细规划，做到控规全覆盖。全面开展市域体系规划编制，做好保障性住房等民生工程规划。落实历史文化名城的保护规划，编制好老城南华门及明太原县城等历史文化街区保护建设实施导则。

　　加快城市基础设施建设。围绕“2020年建成区增加100平方公里、人口增加100万”的总体目标，重点推进以下几方面工程建设：按照“质量一流、速度一流”的要求，完成中博会配套建设工程。加快推进城市道桥建设，实施太行路等44条项道路新建、改造工程。启动轨道交通工程。加快推进城市配套项目建设，加快建设城市供热、供水、供气、供电、市容环卫、污水处理、垃圾处理、景观改造、应急避难场所、防灾减灾等工程项目。加快建设长风商务区等高品位亮点片区。以小街小巷拓宽改造为依托，在每个城区选择1个片区进行高标准集中连片改造。坚持公交优先发展，大力推进公共交通基础设施建设。

　　大力提升城乡管理与服务水平。瞄准“两年大变样、五年创一流”的目标，深入推进“城乡清洁工程”，对市政设施、公用事业等十类重点领域进行集中整治。以打造“数字城管”、“法治城管”、“服务城管”为重点，提高科学化、法制化、精细化管理和服务水平。理顺市区两级城管执法体制，推进事权下放和“市政服务进社区”，健全和完善“两级政府、三级管理、四级网络”的城乡管理新格局。加强出租车经营管理，实现文明、规范服务。

　　加快小城镇及新农村建设。继续组织开展 “省级村庄建设示范镇”创建工作，突出特色镇和中心村建设。以新农村建设统揽“三农”工作，科学确定一批具有代表性的省级新农村建设重点推进村，启动新农村连片建设提升工程。完成县乡公路改造150公里，“村村通”改造100公里。强力推进棚户区、预制板房和农村危房改造。加快农村集中供热、污水处理、垃圾处理等基础设施建设步伐。加强道路两旁等违法违规建设的监管。年内创建5个生态乡镇和10个生态文明村。支持老区发展，加大扶贫力度，全年脱贫1.9万人。

　　积极推进太原都市圈核心区建设。以太榆同城化为重点，拓展太原核心区发展空间。围绕“一核一区三组群”格局和“一小时通达”交通圈要求，加大基础设施建设力度，推进市政设施向清徐、阳曲延伸。推进东环高速公路外移和国道108复线工程建设。加快太原汽车客运南站和东南站建设进度。着手推进太原核心区内公交运营、供水供热等基础设施协调发展，科教、文化、通信、旅游、户籍管理等事项一体化服务。

　　各位代表，第六届“中博会”今年在太原举办，这是我市经济社会发展中的一件大事、喜事。我们将以“迎中博、创环境、争一流”为主题，抓好城乡基础设施的建设完善，抓好市容市貌的综合治理，抓好城乡管理水平的全面提升，以优良的环境、优质的服务、完善的保障，笑迎八方宾客，展示太原新形象！

　　（四）大力实施科教兴市和人才强市战略，在增强自主创新能力上下功夫。

　　建设一流的自主创新基地，科技、教育和人才是基础和根本，必须始终放在重要的战略位置。

　　积极推进科技创新。支持园区、企业提高自主创新能力，与国内外一流的产业对接，与国家重大战略性科技项目对接。搭建科技创新平台，强化产学研联盟，重点建设一批工程技术中心、企业技术中心、企业工程实验室。年内市级以上企业技术中心突破100户，拥有自主知识产权的规模以上企业达到40%。加快高新技术成果转化及产业化，高新技术产业增加值增长18%。重点组织实施中小企业信息化改造、民生科技成果应用转化等五大科技专项行动。专利申请量和授权量均增长30%，经省鉴定的科技成果增长15%，达到国内领先水平以上的成果占65%，科技成果转化率达到55%。构建科技多元化融资体系，加大财政科技投入，全社会研究与试验发展经费投入占地区生产总值的比重达到3%。

　　优先发展教育事业。大力推进义务教育标准化学校建设，提高教育基础设施水平。深化“联盟校”工作，促进优势教育资源公平化。推进课程和教学改革，注重培养学生独立思考、实践创新能力，切实减轻中小学生课业负担。保证中小学生每天一小时校园体育活动。整合中等职业教育学校和专业布局，筹建职业教育综合实训基地。加快太原大学搬迁，推进二期工程建设。完善学校安防工作体系，创建平安校园。规范学校办学行为和教师从教行为，提高教育质量和办学水平。

　　全面加强人才工作。重点抓好“869人才计划”的落实。以转型跨越发展为导向，以高层次和高技能人才为重点，制定引进奖励政策，培养引进一批创新型科技人才和急需紧缺人才。建立健全政府宏观管理、市场有效配置、单位自主用人、人才自主择业的体制机制，努力营造平等公开、竞争择优的制度和尊重人才、服务人才、留住人才的社会环境，激励优秀人才脱颖而出，创造人尽其才的良好局面。

　　（五）着力保障和改善民生，在加快社会发展建设 上下功夫。

　　经济越发展，越要重视保障改善民生和加强社会建设，让人民群众切实享受改革发展成果。

　　着力保障和改善民生。将公共财政支出向民生倾斜，大力解决事关广大群众切身利益的问题。把稳定物价总水平作为今年保障和改善民生的首要任务，加强价格监管，严厉打击哄抬物价、欺行霸市的行为，切实维护市场秩序，全力保障农产品、基本生活必需品、重要生产资料的生产和供应。对养老保险制度未覆盖的城镇居民实施养老保险，今年在全省率先实现养老保险全覆盖。在全省率先推进社会保障“一卡通”。落实最低工资标准。劳动合同签订率达到95%以上。全面推进创业型城市建设，着力解决高校毕业生、困难群体、农民工等群体的就业问题，全市城镇新增就业人数达到9.5万人、困难群体就业达到1.1万人。制定出台征地预存款制度，有效保障被征地农民利益。推进社会救助体系建设，建立临时救助制度，继续提高城乡低保标准，完善低保标准与物价指数上涨联动机制，切实保障低收入群众的正常生活。按照“政府主导、市场运作、整村拆除、安置优先、改善民生”原则，高标准实施城市中心区城中村改造，让城中村居民真正享受到城市化发展带来的成果。启动实施农村新的“五个全覆盖”工程，用两年时间，实现农村街巷硬化全覆盖、农村便民连锁商店全覆盖、农村文化体育场所全覆盖、中等职业教育免费全覆盖和新型农村社会养老保险全覆盖，使广大农民群众得到更多的实惠、过上更加美好的生活！推进残疾人社会保障和服务体系建设，筹建太原市残疾人托养中心。发展慈善事业，开展“慈善一日捐”和慈善救助活动。

　　加强和创新社会管理。深入推进国家社会管理创新试点市工作，“对标”一流，创新社会管理理念、方法和体制，实施十个重点创新项目，确保不发生重大群体事件，不发生重大安全事故；确保各类案件下降，信访总量下降；确保社会管理精细化水平提高，群众满意度上升。加强社区建设，夯实社会管理的基础。充分发挥社区对农民工的服务功能。在3000人以上乡（镇）、村建设20个社区综合服务中心。巩固和完善“管、防、打、控”机制，确保社会治安持续稳定。注重加强城乡防御各种自然灾害的基础设施建设，提升气象灾害、地震灾害监测预警水平。建立健全应急救援体系，提高突发事件应急处置能力。争创“全国双拥模范城”七连冠。统筹解决人口问题。做好第六次人口普查工作。加强民族宗教、档案、妇女儿童、老龄和红十字会等工作。

　　推进医药卫生事业改革发展。今年是医改三年实施方案的攻坚年，要确保完成各项目标任务。实施“百院兴医”工程，加强医疗卫生机构的基础设施建设。继续推进公立医院改革试点，规范发展民营医院。在二级以上医院全面推行济困救助、门（急）诊病历“一本通”、医学检验检查结果“一单通”和预约诊疗服务。社区卫生服务机构建设达标率达到90%。加强医疗机构和药品、医疗器械市场的监管，维护医疗市场秩序和职业卫生、公共卫生安全。建立城镇居民大病医保制度，实现城镇职工医保市级统筹。

　　切实抓好安全生产工作。深入开展“安全生产年”活动，突出抓好煤炭、非煤矿山、尾矿库、道路交通、消防、危险化学品、特种设备、易燃易爆物品、建筑和公共场所的安全专项整治，严厉打击非法违法生产经营建设行为，确保安全生产形势持续稳定好转。大力开展食品安全整顿，建立生产与流通全过程质量监控机制。提高餐饮安全保障水平，创建350家餐饮安全示范店。

　　搞好房地产市场调控。落实房地产市场调控政策，健全长效机制，全力推进保障性住房建设，重点解决城镇低收入家庭住房困难，切实稳定房地产市场价格，满足居民合理住房需求。加强房地产市场监测和市场行为监管，严厉查处各类违规违法行为。

　　各位代表，民生问题是我们一切工作的着眼点和立足点。解决好民生问题，必须更加注重雪中送炭。我们要采取切实有效的措施，让人民群众特别是困难群众真正享受到发展的成果，让他们生活得更加美满！更加幸福！

　　（六）加快改革开放步伐，在增强发展的动力与活力上下功夫。

　　建设一流的省会城市，必须进一步深化改革开放，激发创造活力，构建体制机制支撑。

　　努力深化改革。理顺国资管理体制，突出项目引领，推动国企改革向整合资源、强化管理转变。大力推行节约集约用地，全面推进城乡建设用地增减挂钩工作，率先启动矿业用地改革试点。加强“金融生态”建设，清理规范政府融资平台，完成整合重组。深化财政管理体制改革，完善市县财政体制，严格收入入库级次管理；逐步建立预算编制、执行和监督相分离的管理体制。扩大政府集中采购范围，制定有效措施，实现应采尽采。

　　扩大对外开放。积极抓好引资项目的审批、履约与跟踪服务，提高项目履约率和资金到位率。调整外贸结构，推动出口产品升级，地区外贸进出口总额增长15%。拓展对外经济合作领域，重点打造以经济区为中心的离岸服务区、以高新区为代表的信息产业服务区和以民营区为代表的物流服务外包区，推进我市在服务外包上跨越发展。

　　大力发展民营经济。切实解决影响民营经济发展的各种制约因素，制定出台支持中小企业和民营经济加快发展的政策，建立支持鼓励发展的产业目录和项目库，提供发展平台，创新发展环境。引导民间资本投资优势产业和新兴产业，促进民营经济转型发展。强化金融服务，建立完善担保机构，使民营经济在融资渠道和融资额度上有新的突破。

　　（七）推进民主法制和城市文化建设，在加强政治保障和精神支撑上下功夫。

　　推进民主法制建设。认真执行人大及其常委会的决议决定，自觉接受人大、政协和社会各界的监督，广泛听取各民主党派、工商联、无党派代表人士的意见和建议。保障和支持审计、监察等部门依法实施监督，提高人大代表建议和政协提案的办理质量和水平，建立实施评价制度，提高落实率，以代表和委员是否满意作为衡量办理工作的标准。启动“六五”普法工作，提高全民法制素质。加强基层民主建设，做好第九届村委会换届工作。

　　加快城市文化建设。大力倡导共同的理想追求，培育城市核心价值观。深入推进社会公德、职业道德、家庭美德、个人品德建设，不断提高市民文明素质。广泛开展群众性精神文明创建活动，争创全国文明城市。精心塑造城市文化特色，加大城市形象设计、文化标识等体现城市特质要素的研究和宣传。净化社会文化环境，加强未成年人思想道德和校外活动场所建设，保护青少年身心健康。全面启动明太原县城、南华门等区域的保护和修复，建设一批代表城市演进轨迹的历史街区、景区，彰显太原历史文化名城的独特风貌。做好第四批国家级非物质文化遗产保护项目申报工作，积极筹建太原非物质文化保护中心和非物质文化主题公园。繁荣文化广电新闻出版事业，推出文艺精品。完善公共文化服务体系，继续实施文化惠民工程。大力发展群众体育事业。积极备战全国第七届城市运动会和伦敦奥运会，精心办好第二届太原国际马拉松赛。

　　（八）全面加强政府自身建设，在打造一流发展环境上下功夫。

　　建设一流省会城市，狠抓落实是关键。必须全方位开展对标活动，以推进效能政府建设为突破口，全力建设人民满意政府，倾力打造一流发展环境。

　　优化政务环境。引入第三方咨询服务，以创新政府绩效考核为中心环节，以信息化为手段，深化行政审批制度改革；推进法治政府建设，进一步完善民主科学决策的体制机制，强化重大决策调查研究，实施决策风险评估，充分听取民意，提升决策科学化、民主化水平；推进服务政府建设，在正确履行经济调节、市场监管职能的同时，更加注重社会管理和公共服务，把工作重点放到为市场主体服务上，把功夫下在为经济发展创造良好环境上，着力提供更多更好的公共产品和公共服务。

　　大力提高执行力。全体公务员特别是领导干部要加强学习调研，提高履职能力，通过不断学习增长才干，通过深入调研提高水平，研究新情况，解决新问题。强化纪律意识，对上级的决策部署执行有力，提高办事效率，坚决杜绝推诿扯皮、办事拖拉。要想得细、做得实，把每一件事落到实处。发挥好“三位一体”政务督查的积极作用，以有效的督查促进工作落实。强化行政问责，对失职渎职、不作为和乱作为的，严肃追究责任。

　　加强廉政建设和反腐败工作。推进政府信息公开，规范行政权力运行，坚持勤俭节约，反对铺张浪费。加强廉政教育，严格执行中央和省关于廉洁从政的各项规定，全面落实政府系统党风廉政建设责任制。今年将重点抓五个问题的整治：一是坚决整治领导干部收受礼金问题，明令禁止任何人以各种名义接受服务对象的礼金、各种有价证券和支付凭证；二是坚决整治领导干部公务用车，集中开展公务用车专项治理；三是坚决制止领导干部请吃或吃请，公务接待不得超标准；四是坚决整治领导干部插手经济活动；五是坚决整治领导干部为子女、亲友等谋取利益。

　　各位代表，解决群众最关心、最直接、最现实的利益问题，是人民政府义不容辞的责任，也是广大人民群众的迫切期盼。今年市政府承诺办好以下十件实事：一是加快解决困难群众“住房难”问题，开工新建保障性住房4万套。二是缓解“入学难”、“入园难”问题，新建、改建、扩建项目学校200所、公办幼儿园30所。三是努力缓解“看病难、看病贵”问题，在50个社区设置自助健康体检小屋，在30个乡镇卫生院建立远程诊断系统。四是着力缓解“菜价高”的问题，新增设施蔬菜1.5万亩，构建绿色蔬菜通道，建成标准化菜市场40个。五是改善市民出行条件，加大政府投入，新增公交车辆600台。六是提高农村8万人的安全饮水标准。七是为2400名贫困残疾人提供康复救助，为118个社区（机构）配发残疾人康复训练器材，为260户农村贫困残疾人家庭实施危房改造。八是关爱老人，建立覆盖全市的应急呼叫援助公共服务系统，为生活困难的空巢（独居）老人配发1万部“爱心一键通”。九是解决市民和游客上厕所难问题，新建公共厕所100个。十是实施文化惠民，为全市540个社区文化活动室统一配备文化活动设施器材。

　　各位代表，建设一流省会城市是全市上下的共识，再造一个新太原是全市人民的愿望。让我们在省委、省政府和市委的坚强领导下，抓住机遇、迎难而上、团结奋斗、顽强拼搏，全力以赴推动我市经济社会转型跨越发展，努力实现“十二五”良好开局，以优异的成绩迎接中国共产党建党90周年！
